# Unfolded Protein Response–Related Signature Associates With the Immune Microenvironment and Prognostic Prediction in Osteosarcoma

**DOI:** 10.3389/fgene.2022.911346

**Published:** 2022-06-08

**Authors:** Zhao Zhang, Xincheng Liu, Debin Cheng, Jingyi Dang, Zhenzhou Mi, Yubo Shi, Lei Wang, Hongbin Fan

**Affiliations:** Division of Musculoskeletal Cancer Service, Department of Orthopaedic Surgery, Xi-jing Hospital, The Fourth Military Medical University, Xi’an, China

**Keywords:** unfolded protein response, prognostic prediction, immune infiltration, nomogram, osteosarcoma

## Abstract

**Background:** Osteosarcoma is a highly malignant bone tumor commonly occurring in adolescents with a poor 5-year survival rate. The unfolded protein response (UPR) can alleviate the accumulation of misfolded proteins to maintain homeostasis under endoplasmic reticulum stress. The UPR is linked to the occurrence, progression, and drug resistance of tumors. However, the function of UPR-related genes (UPRRGs) in disease progression and prognosis of osteosarcoma remains unclear.

**Methods:** The mRNA expression profiling and corresponding clinical features of osteosarcoma were acquired from TARGET and GEO databases. Consensus clustering was conducted to confirm different UPRRG subtypes. Subsequently, we evaluated the prognosis and immune status of the different subtypes. Functional analysis of GO, GSEA, and GSVA was used to reveal the molecular mechanism between the subtypes. Finally, four genes (*STC2*, *PREB*, *TSPYL2*, and *ATP6V0D1*) were screened to construct and validate a risk signature to predict the prognosis of patients with osteosarcoma.

**Result:** We identified two subtypes according to the UPRRG expression patterns. The subgroup with higher immune scores, lower tumor purity, and active immune status was linked to a better prognosis. Meanwhile, functional enrichment revealed that immune-related signaling pathways varied markedly in the two subtypes, suggesting that the UPR might influence the prognosis of osteosarcoma *via* influencing the immune microenvironment. Moreover, prognostic signature and nomogram models were developed based on UPRRGs, and the results showed that our model has an excellent performance in predicting the prognosis of osteosarcoma. qPCR analysis was also conducted to verify the expression levels of the four genes.

**Conclusion:** We revealed the crucial contribution of UPRRGs in the immune microenvironment and prognostic prediction of osteosarcoma patients and provided new insights for targeted therapy and prognostic assessment of the disease.

## Introduction

Osteosarcoma is one of the most frequent primary malignant bone tumors in adolescents, often occurring in fast-growing long bones (Tang et al., 2008; Arndt et al., 2012). With significant advances in surgical options, neoadjuvant chemotherapy, and diagnostic imaging, the overall survival of patients with osteosarcoma has been significantly prolonged (Li et al., 2015; Wakamatsu et al., 2019; Hao et al., 2021). Nevertheless, the outcome of metastatic and recurrent patients remains unsatisfactory due to its intensely aggressive feature, with a 5-year mortality ratio of below 20% (Ferrari et al., 2005). In addition, high tumor heterogeneity, leading to chemoresistance in some patients, remains challenging clinically (Xiao et al., 2015). Therefore, there is a need for the discovery of novel targets for targeted treatment and better prognostic markers for osteosarcoma.

The unfolded protein response (UPR) is a highly conserved homeostatic response in all eukaryotic cells and can help cells mitigate the accumulation of misfolded proteins in the endoplasmic reticulum (ER) (Hetz et al., 2020). Under ER stress conditions, such as hypoxia, nutritional deprivation, acidosis, and inflammatory stimuli, the UPR can activate three sensor proteins (IRE1α, PERK, and ATF6) to initiate specific signal transduction cascades which regulate the rate of protein production for maintaining ER homeostasis (Vanacker et al., 2017). Lately, extensive studies have indicated that the UPR acts a dual function in tumor occurrence and development. In earlier stages of tumorigenesis, the UPR is capable of exerting antitumor effects to hinder tumor transformation, while in the established tumors, tumor cells can induce chronic UPR to relieve ER stress-induced apoptosis and develop drug resistance to maintain tumor survival (Ma and Hendershot, 2004; Yang et al., 2019). Aberrant activation of the UPR was found in a wide range of tumors, including bladder cancer, cutaneous melanoma, and liver cancer (Houessinon et al., 2016; Wan et al., 2020; Zhu et al., 2021). Apart from a direct impact on tumor biology, the UPR also has the ability to remodel the tumor immune microenvironment (TIME) to regulate the crosstalk between immune cells, which serves an essential function in immune surveillance and immune escape (Zanetti et al., 2022). Mahadevan et al. demonstrated that the UPR can modulate the phenotype of dendritic cells and CD8^+^ T cells to facilitate tumor growth. Notably, previous studies have confirmed the UPR to be relevant to the growth, prognosis, and drug resistance of osteosarcoma (Mahadevan et al., 2012; Yan et al., 2015; Shi et al., 2022). However, the role of the UPR-related gene (UPRRG) sets on prognostic prediction and immune infiltration in patients with osteosarcoma remains unclear.

Recently, following the advancement of the high-throughput sequencing technology for tumor genomics, probing new molecular patterns through bioinformatics approaches offers new insights for tumor treatment and prognosis evaluation (Qian et al., 2021). In the present study, we performed a comprehensive analysis of the prognosis and immune microenvironment of different molecular subtypes based on the expression of UPRRGs and revealed potential functional and signaling pathways. Moreover, we developed and validated a novel prognostic signature for predicting the prognosis of osteosarcoma patients *via* integrating risk scores and clinical features.

## Methods and Materials

### Data Collection

The mRNA expression profiles and corresponding clinical features of 85 osteosarcoma patients were acquired from the TARGET database as a training cohort (https://ocg.cancer.gov/programs/target). UPRRGs were extracted from hallmark gene sets from the Molecular Signature Database (Zhang et al., 2021). In addition, mRNA expression profiles and relevant clinical information of 53 osteosarcoma patients from GSE21257 were obtained from the GEO database to serve as an external validation cohort (https://www.ncbi.nlm.nih.gov/geo/). The clinical information for osteosarcoma patients is presented in Supplementary Table S1.

### Classification of Molecular Subtypes

To assess the biological functions of UPRRGs in OS, we first identified 15 prognosis-related UPRRGs for osteosarcoma based on a univariate Cox regression analysis. Subsequently, consensus clustering analysis was conducted based on the expression matrix of these 15 genes with the R package “ConsensusClusterPlus”. For the analysis, 80% of the total samples of the target dataset are included in each iteration and reiterated 1,000 times to ensure cluster stability. The average consistency value and clinical significance within the clustering group were used together with the optimal number of clusters. Principal component analysis (PCA) was performed to confirm the gene expression patterns in the different clusters. KM curve and log-rank tests were employed to evaluate the prognosis of different clusters. The heat map was used to show the gene expression and clinical characteristics of the different clusters.

### TIME Evaluation

The tumor microenvironment scores for individual samples in the different subtypes were assessed by the ESTIMATE algorithm (Yoshihara et al., 2013). The TIMER algorithm was conducted to comprehensively evaluate the abundance of immune infiltrating cells in each sample (Li et al., 2020). Moreover, the infiltration abundance of 28 immune cell types for an individual sample was tested by the single sample gene set enrichment analysis (ssGSEA) algorithm. The expression of immune checkpoint (ICP) genes in different subtypes was also evaluated.

### Identification of DEG and Enrichment Analysis

Differentially expressed genes (DEGs) in different subtypes were analyzed *via* the R package “limma”, and log2 (Foldchange) > 1.5 and FDR<0.05 were considered as the threshold. Gene Ontology (GO) terms of DEGs were enriched by using the R package “clusterProfiler” and visualized *via* Metascape (https://metascape.org) (Zhou et al., 2019). Gene set variation analysis (GSVA) was conducted to verify functional pathway variations between the different subtypes according to GO: the biological process (BP). Also, Gene Set Enrichment Analysis (GSEA) was employed to analyze hallmark gene sets for different subtypes (Subramanian et al., 2005). |NES| > 1, NOM p-val < 0.05, and FDR<0.25 were taken as the threshold.

### Establishment and Validation of the Prognostic Signature

The previously obtained 15 prognosis-related UPRRGs were screened *via* least absolute shrinkage and selection operator (LASSO) Cox regression based on the R package “glmnet”, and the minimum lamba is taken as the optimal value. Then, a multivariate Cox analysis was conducted to further optimize and establish the prognostic signature. The risk score in the training and validation cohorts was calculated with the following formula: Risk score = ∑^i^_n_ (Coef_i_ * X_i_). In the training cohort, all patients were classified into low- and high-risk groups by the median risk score, and the overall survival (OS) between the two groups was investigated using the KM curve and log-rank tests. In addition, receiver operating characteristic (ROC) curves were applied to measure the effectiveness of prognostic models. Furthermore, the abovementioned formula was also used in patients from GSE23257 to generate a risk model to validate the prognostic benefit. Finally, we integrated different clinical characteristics including gender, age, metastatic status, and disease site to assess if the risk score was an independent prognostic element in osteosarcoma patients based on univariate and multivariate Cox regression.

### Construction and Calibration of the Nomogram

A nomogram model was established to forecast the 3-year and 5-year survival probability of osteosarcoma patients *via* integrating risk scores and clinical profiles. The C-index, ROC curves, and calibration plots were employed to survey the predictive performance of the constructed nomogram in both cohorts.

### Patient Sample Collection and Cell Culture

A total of six osteosarcoma patient tissues and corresponding adjacent normal tissues were obtained from the operation and stored in liquid nitrogen banks. The study was approved by the institutional review board of Xijing Hospital, the Fourth Military Medical University, and all patients provided signed informed consent. Moreover, human osteosarcoma cell lines HOS and MG-63 were procured from Procell Life Science & Technology Co. Ltd and cultured in DMEM medium (Gibco, Shanghai, China) containing 10% fetal bovine serum (FBS; Gibco, Shanghai, China) and 1% (v/v) penicillin/streptomycin (Gibco, Shanghai, China). The osteoblast cell line hFOB 1.19 was provided by Dr. Jianping Bai of Xijing Hospital and cultured in DMEM/F12 medium with 10% FBS, 0.3 mg/ml G418 (Procell, Wuhan, China), and 1% (v/v) penicillin/streptomycin. At 37 C, 5% CO_2_ environment, all cell lines were incubated.

### Quantitative Real-Time PCR (qRT-PCR)

The TRIzol method was utilized to extract and purify RNA from tissues and cells. Then, a cDNA synthesis kit (Takara, China) was applied to reverse transcribe the RNA. The TB Green Premux Ex TaqⅡ (Tli RNaseH Plus) was used for qRT-PCR with the Bio-Rad CFX96 Real-Time PCR system (Bio-Rad, USA). The internal control was GADPH. The primer sequences of the candidate genes are shown in Table 1, and the analysis was conducted three times for all genes.

**TABLE 1:** Primer sequences of the candidate genes.

Gene	Sequence (5' -> 3′)
STC2	Forward:GGGTGTGGCGTGTTTGAATG
	Reverse:TTTCCAGCGTTGTGCAGAAAA
TSPYL2	Forward:ACAGGTGCTGGCCGATATG
	Reverse:CCGACTCGATGGTAGAATCCC
PREB	Forward:ACGGGCCACCATGAACTTG
	Reverse:GGGTTTCCGCTCCACATTTCT
ATP6V0D1	Forward:TTCCCGGAGCTTTACTTTAACG
	Reverse:CAAGTCCTCTAGCGTCTCGC
GAPDH	Forward:GGAGCGAGATCCCTCCAAAAT
	Reverse:GGCTGTTGTCATACTTCTCATGG

### Statistics

R software (version 4.0.5), SPSS 21.0 software, and GraphPad Prism 8 were carried out for all statistical analyses. The *t*-test was applied for two groups. One-way ANOVA was applied to three groups. *p* < 0.05 was taken as statistically significant. ∗*p* < 0.05; ∗∗*p* < 0.01; and ∗∗∗*p* < 0.001.

## Results

### Identification of UPRRG Molecular Subtypes via Consensus Analysis

**FIGURE 1:**
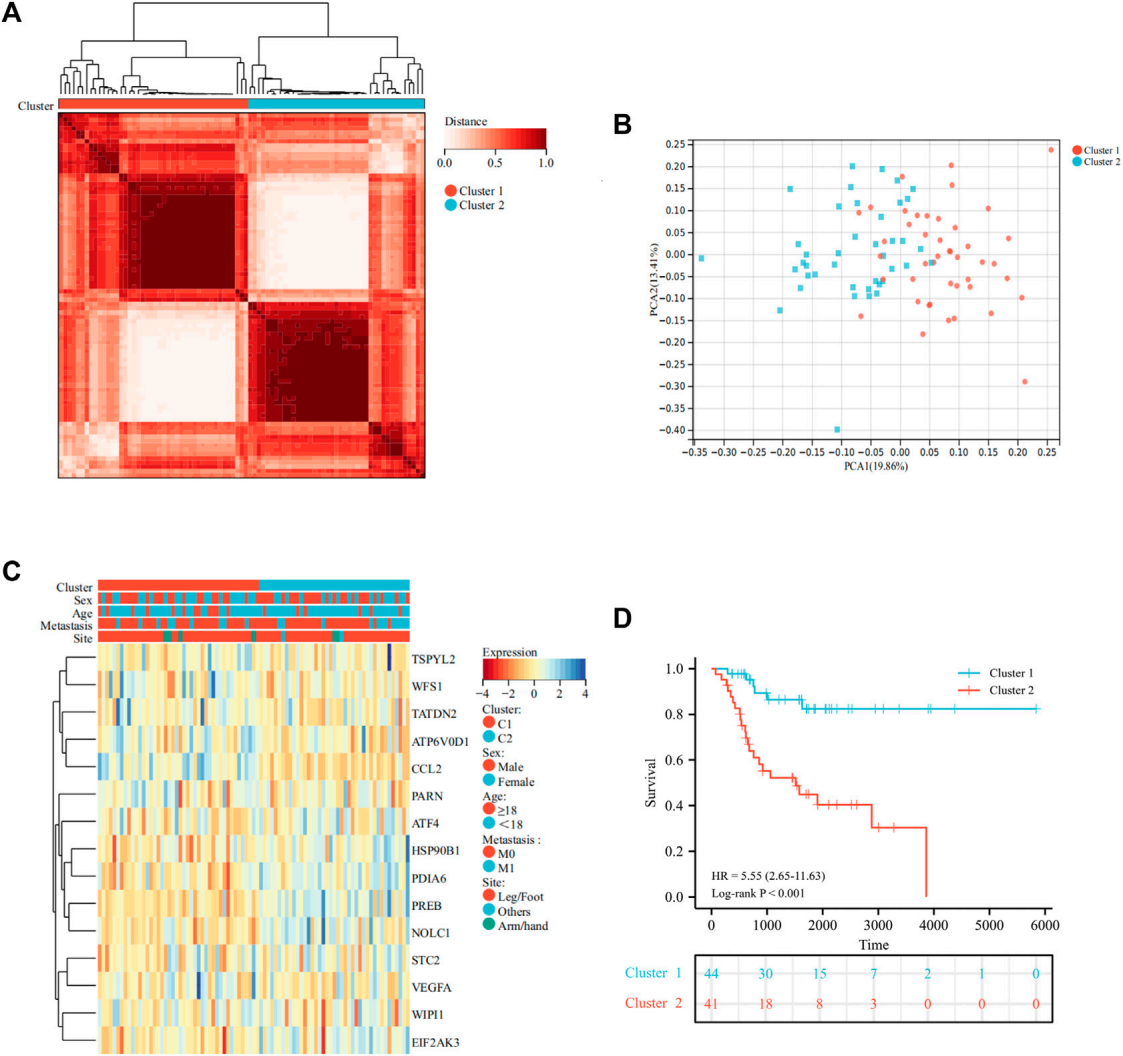
Identification of molecular subtypes of UPRRGs by consensus clustering. **(A).** Clustering heat map at k = 2. **(B)** PCA plot between the two subtypes. **(C)** Heat map of the UPR-related gene expression and clinical features in the two subtypes. **(D)** Survival curves for the two subgroups.

**FIGURE 2:**
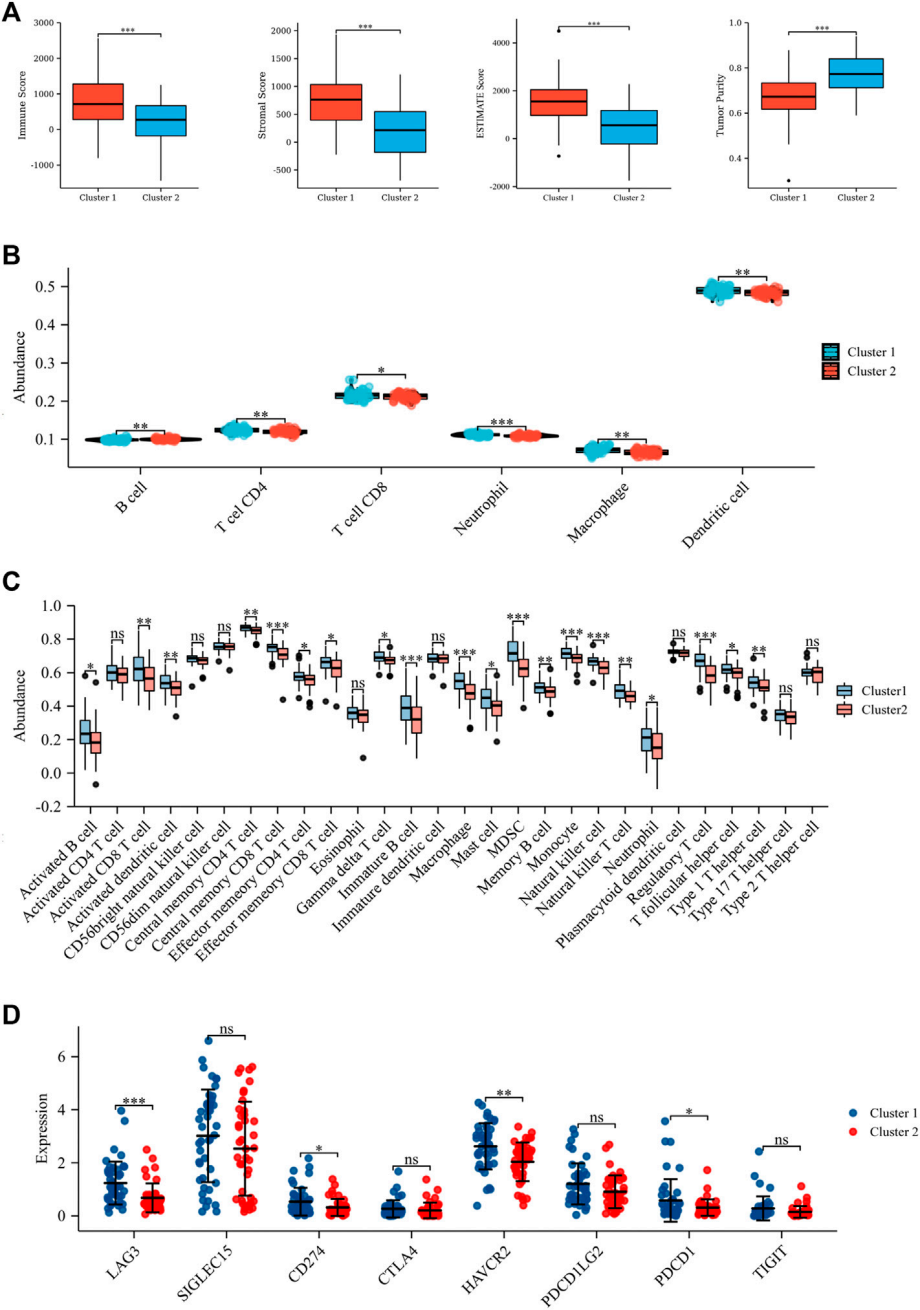
Tumor microenvironment in the two subtypes. **(A)** Stromal score, immune score, ESTIMATE score, and tumor purity based on ESTIMATE algorithm. **(B)** Six immune cell abundance assessments by the TIMER algorithm. **(C)** Twenty-nine immune cell abundance assessments by the ssGSEA algorithm. **(D)** Differences in immune checkpoints between the two subtypes. *p < 0.05; **p < 0.01; ***p < 0.001.

A total of 113 UPRRGs were retrieved from hallmark gene sets, out of which we identified 15 prognosis-related UPRRGs for osteosarcoma, according to univariate COX analysis (Supplementary Table S2, 3). According to the expression profile of these genes, consensus clustering analysis was used to ascertain subgroups of osteosarcoma patients in the training database. k = 2 is considered the best category number of clusters, depending on the average consistency value and clinical significance within the clustering group (Figure 1A and Supplementary Figure S1). PCA analysis revealed a relatively apparent distinction between the two subtypes (Figure 1B). The heat map illustrated the gene expression profile and clinical characteristics of the two subtypes (Figure 1C). Moreover, we noticed that patients in cluster 2 experienced a dismal prognosis to that in cluster 1 (*p*<0.001, Figure 1D). Previous studies have proven that the UPR can coordinate the crosstalk between immune cells and tumor cells in the TIME to exert immunosurveillance and immunosuppressive functions to influence tumor prognosis (Vanacker et al., 2017). Thus, we then evaluated the differences in the TIME across different subtypes. The ESTIMATE algorithm indicated that cluster 1 had higher immune score (*p* = 8.4e-5), stromal score (*p* = 8.1e-7), ESTIMATE score (*p* = 9.6e-7), and lower tumor purity (*p* = 1.1e-6), as compared to cluster 2 (Figure 2A). In addition, the TIMER algorithm discovered that the abundance of most immune infiltrating cells in cluster1 was significantly increased than in cluster 2, including dendritic cells, neutrophil cells, CD4^+^ T cells, neutrophil cells, and CD8^+^ T cells, while B cells showed the opposite result (Figure 2B). As shown in Figure 2C, ssGSEA analysis found that the abundance of 20 immune cell types was significantly increased in cluster 1 compared to cluster 2. In addition, we also observed that *CD274*, *LAG3*, *HAVCR2*, and *PDCD1* were expressed at an elevated level in cluster 1 than in cluster 2 (Figure 2D). Our findings suggested that the prognosis of different subtypes may be affected by the TIME.

### Functions and Pathway Annotations of DEGs for UPRRG Subtypes

**FIGURE 3:**
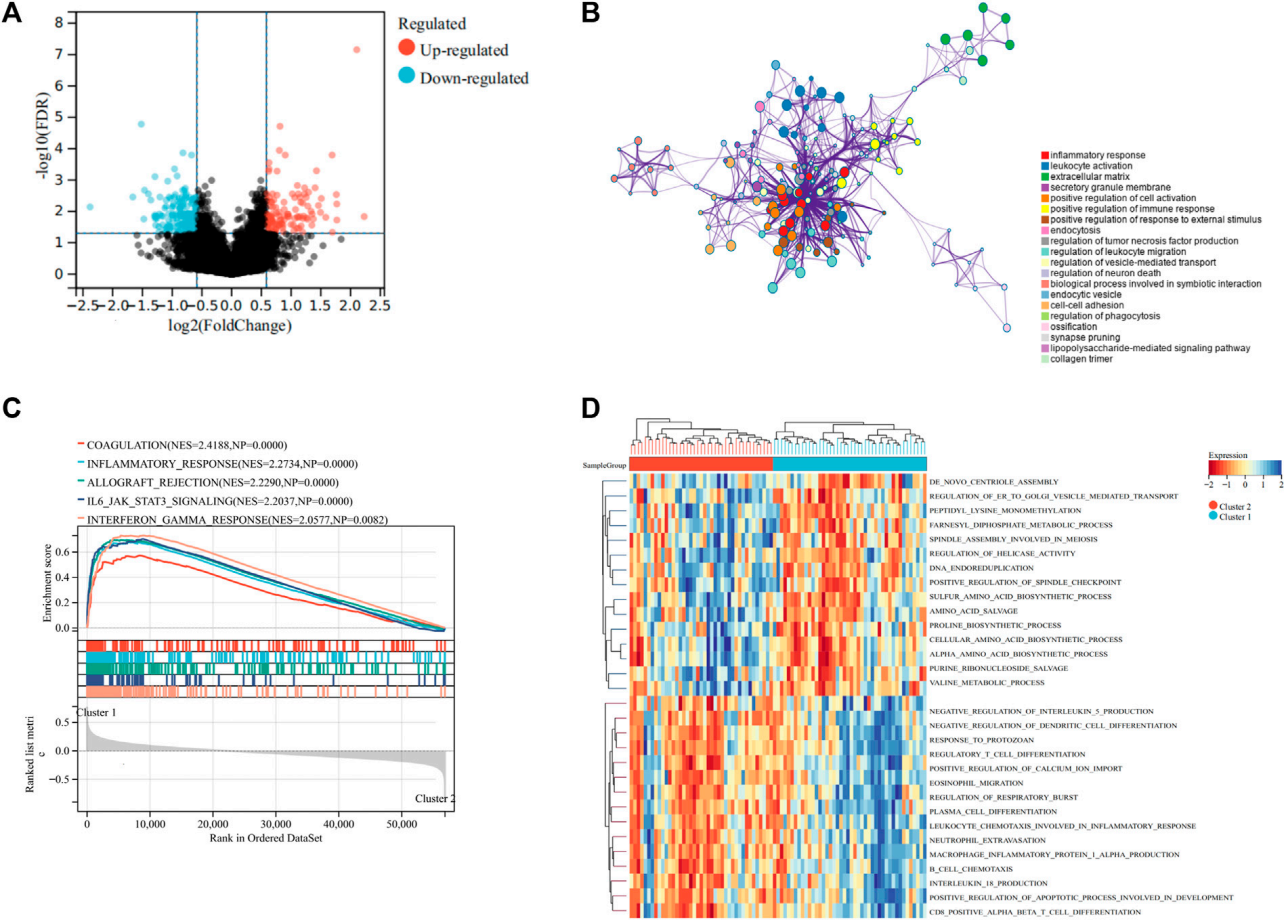
Differentially expressed genes and functional enrichment analyses. **(A)** Volcano plot showing the DEGs between the two subgroups. **(B)** Bubble plot exhibited the functional enrichment of DEGs through GO analysis. **(C)** GSEA shows the hallmark gene sets in the two subgroups. **(D)** Heat map depicted the results of the GSVA analysis.

To reveal the potential mechanisms regulating the TIME between different subtypes, we performed DEGs analysis on the two clusters. The results indicated that 121 genes were upregulated and 157 genes were downregulated in cluster 2 to cluster 1 (Figure 3A). GO analysis implied that these DEGs were primarily associated with inflammatory response, leukocyte activation, and extracellular matrix (Figure 3B). These findings implied that UPR could influence the TIME and prognosis of osteosarcoma *via* modulation of immune-associated pathways. We then used GSVA and GSEA analysis to explore the functional differences in the two clusters. GSEA analysis demonstrated that coagulation, inflammatory response, and IL6/JAK/STAT3 signaling were markedly upregulated in cluster 1 (Figure 3C). GSVA analysis showed that positive regulation of calcium ion import, regulation of the apoptotic process involved in the development, and some immune-related pathways were significantly upregulated in cluster 1 to cluster 2 (Figure 3D). Therefore, we speculated that the UPR plays an essential role in regulating the immune function, thus contributing to the prognosis of osteosarcoma.

### Construction and Validation of the UPRRG Risk Signature

**FIGURE 4:**
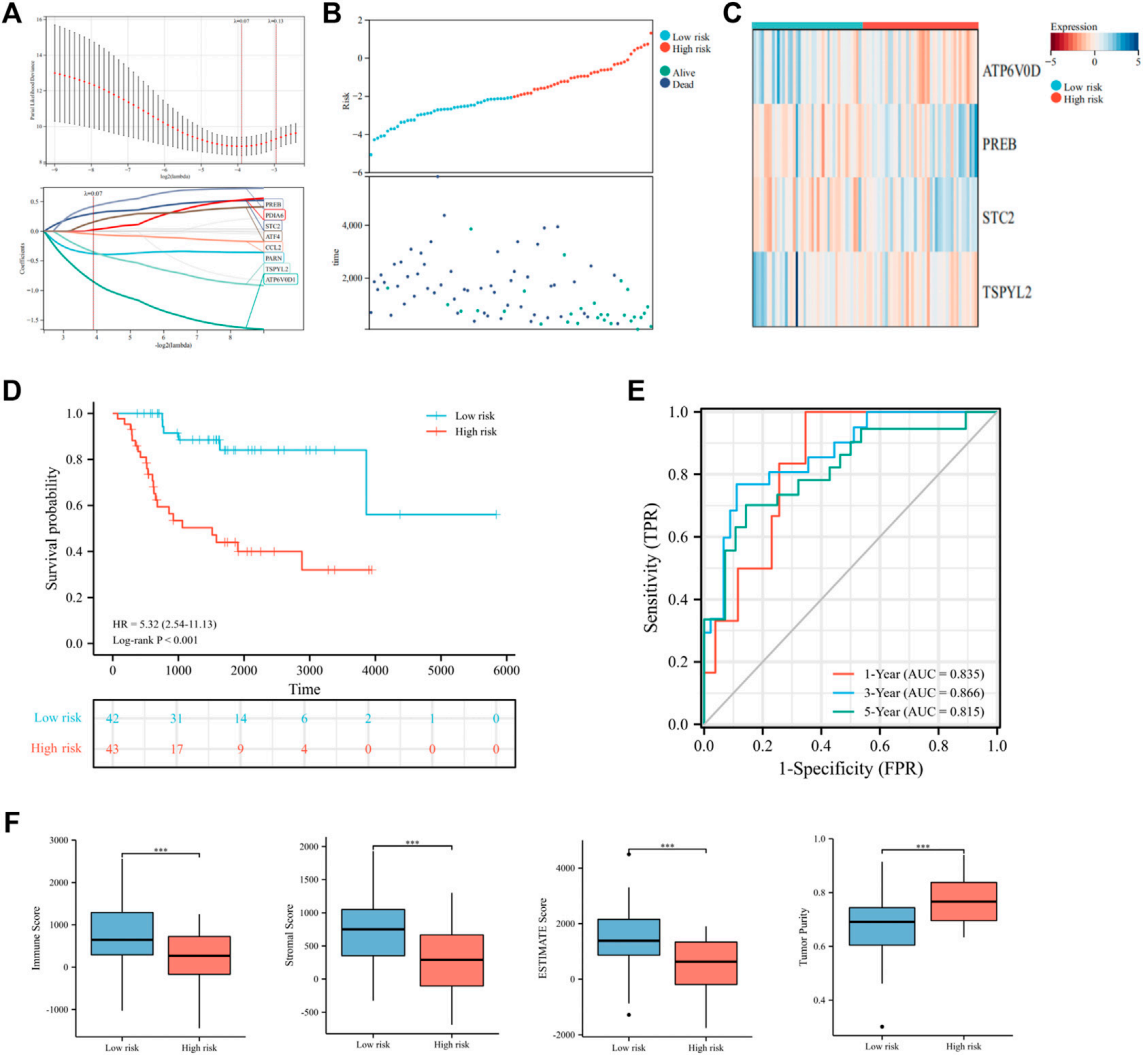
Construction of the risk signature based on UPRRGs in the training cohort. **(A)** Eight optimal UPRRGs filtered by LASSO analysis. **(B)** Distribution of risk scores and patient status in the two risk groups; **(C).** Heat map showing the expressions of four candidate genes. **(D)** Survival curves for the two risk groups. **(E)** Time-dependent ROC curve of the risk model. **(F)** Tumor microenvironment analysis in the two risk groups through the ESTIMATE algorithm. *p < 0.05; **p < 0.01; ***p < 0.001.

**FIGURE 5:**
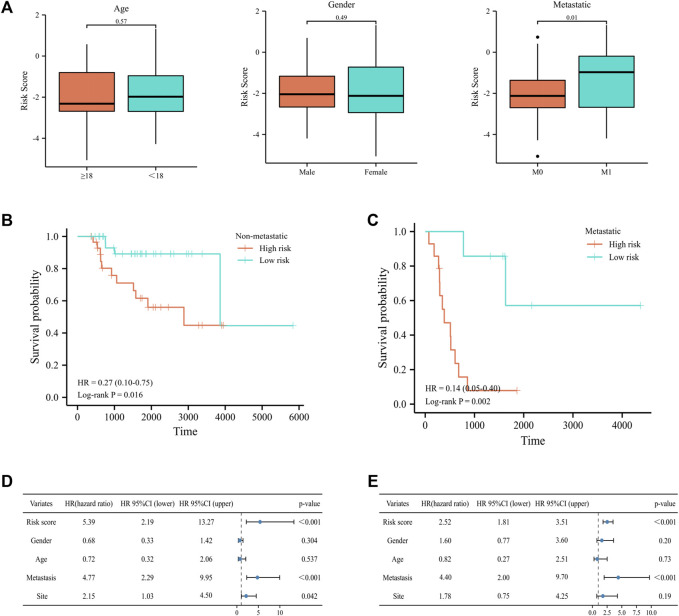
Correlation of the risk signature with clinical features in the training cohort. **(A)** Differences in risk scores among osteosarcoma patients by age, gender, and metastatic status. **(B–C)** Survival curves for patients with osteosarcoma regrouped by metastatic status. **(D–E)** Univariate and multivariate cox regression analyses for integrating risk characteristics and clinical features.

To further elucidate the prognostic predictive role of UPRRGs in osteosarcoma patients, LASSO regression analysis was applied to screen for potential genes, and eight genes were identified by the minimal lambo value (Figures 4A,B). Finally, based on the results of previous screening, four UPRRGs (*STC2*, *PREB*, *TSPYL2*, and *ATP6V0D1*) were identified to establish a risk model for osteosarcoma *via* the multivariate Cox regression analysis (Figure 4C and Supplementary Table S4). The following formula was applied to generate the risk score for each sample: Risk score = -1.523 × ATP6V0D1 + 0.903× PREB +0.586 × STC2 -0.760 × TSPYL2. All patients in the training cohort were classified into high- and low-risk groups by the median risk scores, and patients in the high-risk group showed shorter survival intervals than those in the low-risk group (Figure 4D). KM curves demonstrated a poor prognosis in the high-risk group to the low-risk group (Figure 4E). Moreover, time-dependent ROC analysis found the AUC values of 1, 3, and 5 years were 0.84, 0.87, and 0.83, respectively, which suggested our risk signature showed excellent predictive performance (Figure 4F). Notably, we also observed that the low-risk group saw higher immune scores (*p* = 3.5e-4), stromal scores (*p* = 1.1e-4), ESTIMATE scores (*p* = 3.3e-5), and lower tumor purity (*p* = 2.9e-5) relative to the high-risk group, which suggested that the TIME might be strongly linked to prognosis in different risk groups (Figure 4G). Then, the relevance between the risk signature and clinical features was also evaluated, and the results revealed metastatic patients had significantly higher risk scores than non-metastatic patients (*p* = 0.03), while no differences were found in any other clinical characteristics (Figure 5A). When patients were reclassified for metastatic status, there was a significantly improved prognosis for patients in the low-risk group over those in the high-risk group (Figures 5B,C). These findings supported that the UPRRG risk signature might be strongly correlated with the metastasis status in patients with osteosarcoma. Furthermore, multivariate Cox analysis demonstrated that the risk score and metastatic status were independent prognostic factors for osteosarcoma patients, which meant that the UPRRGs risk model was applicable to predict survival in osteosarcoma (Figures 5D,E).

**FIGURE 6:**
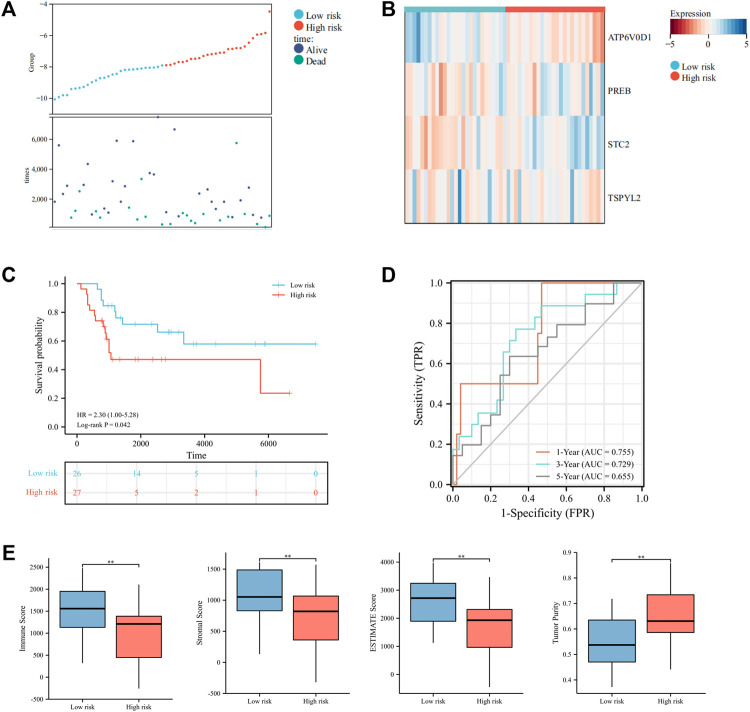
Validation of the constructed risk signature in the verification cohort. **(A)** Distribution of risk scores and patient status in different risk groups. **(B)** Heat map displayed the expressions of four candidate genes in the verification cohort. **(C)** Survival curves of the two risk groups. **(D)** Time-dependent ROC curve in the verification cohort. **(E)** Tumor microenvironment analysis by the ESTIMATE algorithm. *p < 0.05; **p < 0.01; ***p < 0.001.

Moreover, we further tested the prognostic performance of the UPRRG risk signature in a validation cohort. As shown in Figure 6A, the patients in the validation cohort were clearly separated into different risk groups *via* the abovementioned formula. The heat map demonstrated these four genes’ expression profiles in subgroups (Figure 6B). The KM curve likewise showed that the low-risk group had a better prognosis (*p* = 0.04) (Figure 6C). ROC curves suggested the risk signature had better prediction accuracy at 1 and 3 years (Figure 6D). Similarly, the ESTIMATE algorithm obtained results consistent with the training cohort, which further confirmed the role of the TIME in the UPRRG risk signature (Figure 6E).

### Construction and Validation of the Nomogram Prediction Model

**FIGURE 7:**
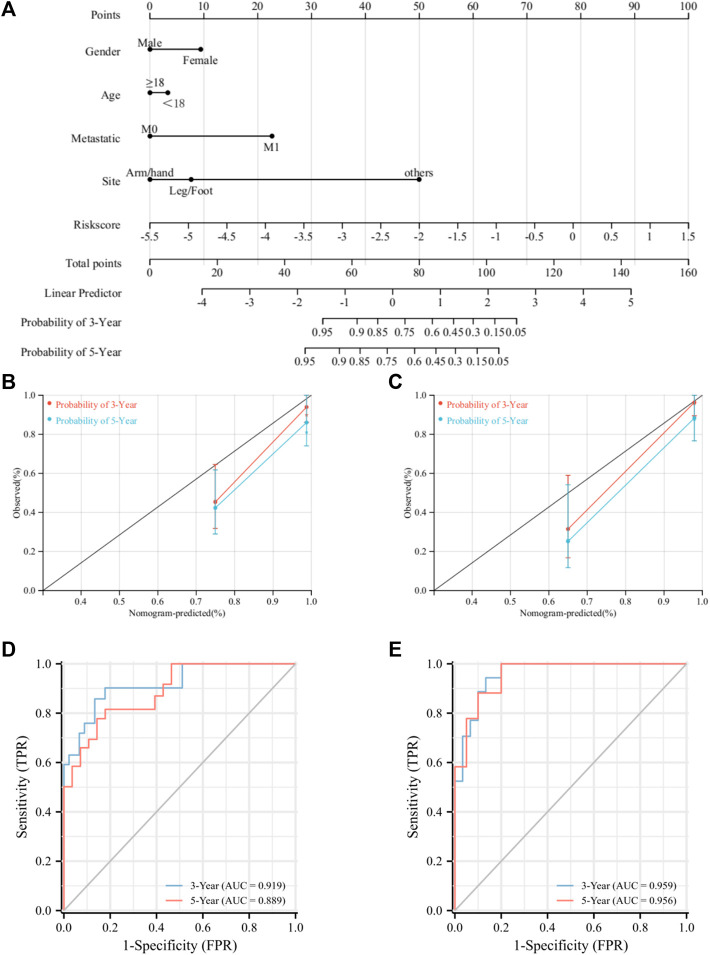
Construction and evaluation of the nomogram. **(A)**. Nomogram for predicting the prognosis of patients with osteosarcoma. **(B)** Calibration for 3-and 5-year OS in the training cohort. **(C)** Calibration for 3-and 5-year OS in the verification cohort. **(D)** ROC analysis for 3-and 5-year OS in the training cohort. **(E)** ROC analysis for 3-and 5-year OS in the verification cohort.

A nomogram model was constructed to improve the accuracy of predicting the prognosis of osteosarcoma patients at 3 and 5 years by integrating risk scores and clinical characteristics (Figure 7A). Then, we validated the predictive efficacy of the nomogram model in the two cohorts. The C-index values for the training and validation cohorts were 0.88 and 0.87, respectively. The ROC curves revealed AUC values of 0.93 and 0.90 for the training cohort at 3 and 5 years, respectively, and for the validation cohort, ROC curves also exhibited excellent prediction accuracy (Figures 7B,C). Moreover, the calibration curves for the training and validation cohort showed that the nomogram model has a strong predictive capacity for the prognosis of osteosarcoma patients at 3 and 5 years (Figures 7D,E). Collectively, the aforementioned results pointed out that the UPRRG nomogram model has high predictive accuracy and can be applied to predict the prognosis of osteosarcoma patients.

### Verification of Candidate Genes by qRT-PCR Analysis

**FIGURE 8:**
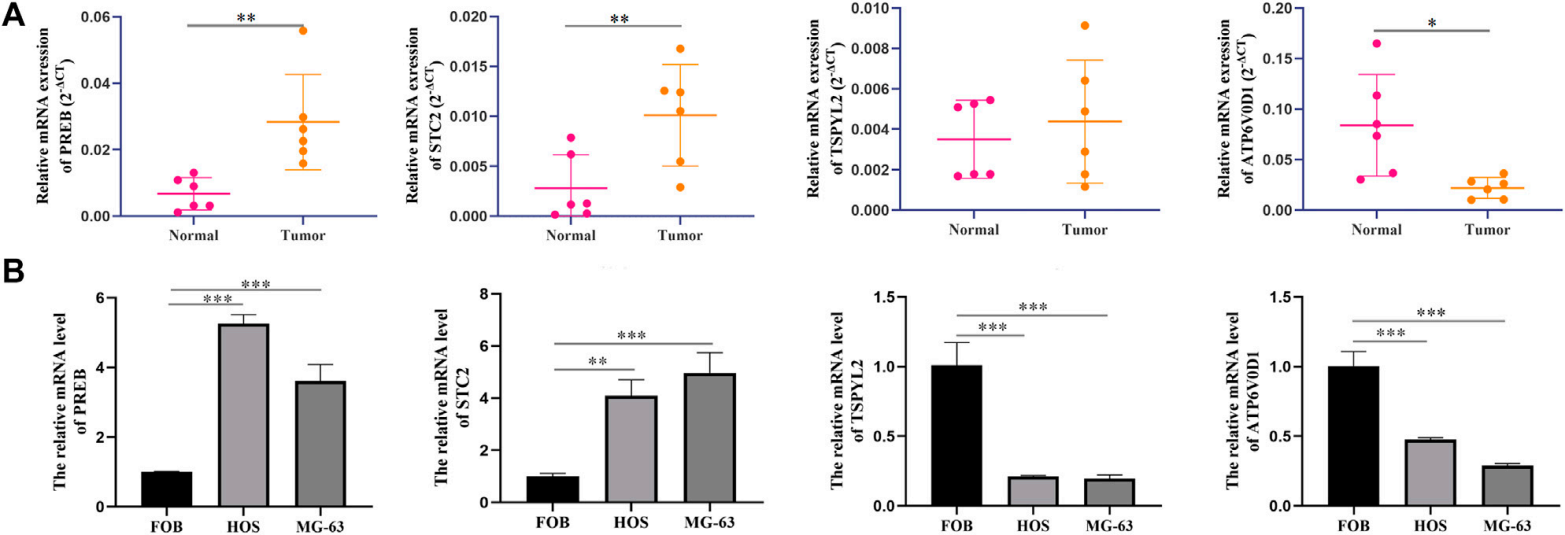
Candidate gene validation. **(A)** Expression levels of candidate genes in tumor and normal tissues from osteosarcoma patients. **(B)** Expression levels of candidate genes in different cell lines. *p < 0.05; **p < 0.01; ***p < 0.001.

To certify the expression levels of these four candidate genes, we performed a qPCR analysis in patients' tissue and cell lines. The results revealed that the expression levels STC2 and PREB were elevated clearly in osteosarcoma tissues than in normal tissues, whereas the ATP6V0D1 expression level was significantly downregulated in tumor tissues compared with normal tissues (Figure 8A). The results of the cell further identified that the expression levels of PREB and STC2 were higher in osteosarcoma cells than in normal osteoblasts, while TSPYL2 and ATP6V0D1 exhibited the opposite results (Figure 8B).

## Discussion

The unfolded protein response is an adaptive signaling pathway that regulates the endoplasmic reticulum homeostasis (Hetz et al., 2020). The UPR has been extensively studied in tumorigenesis, and its abnormal activation is involved in various stages of tumorigenesis and progression (Ma and Hendershot, 2004). Chaiyawat et al. revealed that plenty of UPR-related proteins were highly expressed in osteosarcoma patients and closely associated with chemoresistance, suggesting that targeting UPR pathways might be promising for the treatment of osteosarcoma (Chaiyawat et al., 2019). Ji et al. found that PERK could induce autophagy in osteosarcoma by inhibiting the mTORC1 pathway to counteract ER stress-induced cell apoptosis (Ji et al., 2015). Yan et al. demonstrated that the UPR could inhibit cisplatin-mediated apoptosis by triggering the NF-κB pathway, contributing to drug resistance in osteosarcoma (Yan et al., 2015). Shi et al. identified aberrant activation of UPR-related pathways in osteosarcoma and built a prognostic model for differentially expressed genes (Shi et al., 2022). Although previous studies have shown the UPR was implicated in the development, progression, and treatment of osteosarcoma, the function of UPRRGs on immune infiltration and prognostic prediction in patients with osteosarcoma is not clear.

In this research, we determined two molecule subtypes of osteosarcoma based on prognosis-related UPRRGs and presented the relevance of different subtypes to clinical profiles. Our study found that different subtypes had different survival intervals and TIME. The TIME consists of various components, including immune and stromal cells, which together contribute to tumorigenesis, progression, and prognosis (Zhao et al., 2018). The UPR is a critical factor in regulating the balance of immune dynamics in the tumor microenvironment which can directly affect innate and adaptive immune responses to participate in tumor progression (Martins et al., 2016). Our results indicated that cluster 1 has a better prognosis with higher immune and stromal scores compared to cluster 2. Previous studies have demonstrated that a higher immune score and stromal score were connected to a better prognosis in osteosarcoma, which is in agreement with our findings (Qian et al., 2021). Further analysis indicated that the abundance of multiple immune infiltrating cells was distinctly greater in cluster 1 than in cluster 2, according to the TIMER, indicating that the active immune status of the UPRRG subtypes might be closely better associated with osteosarcoma prognosis. ssGSEA analysis found that the abundance of a variety of tumor-infiltrating lymphocytes was expressed at obviously higher levels in cluster 1 than in cluster 2. Previous studies have demonstrated that the UPR acts as a critical mediator of tumor immunity as an appropriate UPR can induce immune cells to eliminate tumor cells, whereas the sustained UPR could induce immune cell apoptosis to enhance tumor proliferation and invasion (Vanacker et al., 2017). Hence, we believed that the elevated abundance of multiple TILs in cluster 1 might be due to appropriate UPR, which induced activation of TILs to deliver anti-tumor immunity. Nevertheless, the sustained UPR led to massive TIL exhaustion, causing a poor prognosis in patients with osteosarcoma in Cluster 2. Moreover, we also noted that as a consequence of a large number of immune infiltration cells in cluster 1, the expressions of several ICPs were correspondingly elevated in cluster 1 compared to Cluster 2, implying that patients in cluster 1 might be more sensitive to ICP inhibitors. These findings suggested that the identification of UPRRG subtypes may provide a new clinical strategy for prognostic evaluation and targeted therapy of osteosarcoma.

To reveal the molecular mechanisms underlying regulating TIME between different UPRRG subtypes, we carried functional enrichment analysis of the DEGs between the two subtypes. GO analysis suggested that the DEGs were mainly participating in immune-related pathways including inflammatory response and leukocyte activation. In addition, GSEA analysis also confirmed significant enrichment of some immune-related pathways in cluster 1, including coagulation, inflammatory response, and IL6/JAK/STAT3 signaling. The UPR is proven to not only affect the growth and survival of tumor cells but also play an essential role in remodeling the TIME (Zanetti et al., 2022). Batista et al. identified that the UPR participates in the macrophage polarization in the TIME by activating the IRE1α/XBP1 axis, resulting in upregulation of IL-6, IL-23, arginase 1, CD86, and PD-L1 that lead to local immune dysregulation (Batista et al., 2020). Zanetti et al. showed that tumor cells regulate immune cell phenotypes through UPR activation of dendritic cells and T cells to promote tumor proliferation (Mahadevan et al., 2012). In addition, Medel et al. also demonstrated that dendritic cells could enhance CD8^+^ T-cell–specific responses through activation of the IRE1α/XBP1 axis to exert an anti-tumor effect (Medel et al., 2018). Taken together, these findings revealed that the UPR can modulate immune cell infiltration through various immune signaling pathways to improve the prognosis of patients with osteosarcoma. Moreover, GSVA analysis revealed that aside from immune-related pathways, apoptosis and calcium homeostasis-related pathways were also upregulated in cluster 1. These results suggested that the UPR may exert antitumor effects through multiple pathways between UPRRG subtypes. Overall, our data showed that patients with osteosarcoma can activate the UPR in the presence of ER stress to modulate tumorigenesis and progression through multiple pathways and that targeting UPR might be a promising treatment strategy for osteosarcoma.

Next, to assess the role of UPRRGs in predicting the prognosis of osteosarcoma, we constructed a prognostic signature to predict the survival of osteosarcoma patients *via* four genes (*STC2*, *PREB*, *TSPYL2*, and *ATP6V0D1*). Our results found that high expressions of *STC2* and *PREB* were linked to high risk (risk factors), whereas high expressions of *TSPYL2* and *ATP6V0D1* were linked to low risk (protective factors). *STC2* encodes a glycoprotein that performs an essential function in the development and invasion of multiple tumors (Li S. et al., 2021). Previous studies have demonstrated endoplasmic reticulum stress could activate PERK-ATF4 to induce the upregulated expression of *STC2* to inhibit cell apoptosis (Ito et al., 2004). Chen et al. found that *STC2* could promote tumor proliferation by activating the AKT-ERK pathway, and increased STC2 was strongly correlated with poor prognosis in colorectal tumors (Chen et al., 2016). *PREB* can encode transcription factors that bind and activate the basal prolactin promoter activity. Murao et al. showed that *PREB* could act as a transcription factor for TNF-αand IL-1β to regulate the expression of monocyte chemoattractant protein-1, suggesting that *PREB* plays an active role in immune responses (Murao et al., 2009). In addition, *PREB* is a member of a eukaryotic family of WD-repeat proteins involved in many biological activities, including vesicle trafficking, RNA processing, and signal transduction (Taylor Clelland et al., 2000). *TSPYL2*, a member of the TSPY-L nucleosome assembly protein-1 superfamily, can exert anti-tumor effects by inhibiting the cell cycle and regulating DNA damage. Previous studies have found that *TSPYL2* maintains the G1 checkpoint function by inducing p21 transcription to modulate DNA damage and inhibit cellular growth (Tao et al., 2011). Liu et al. indicated that *TSPYL2* could inhibit SIRT1-mediated FOXO3 deacetylation to reduce gefitinib resistance and inhibit DNA damage, implying that *TSPYL2* is a promising therapeutic target (Liu et al., 2022). ATP6V0D1 is an encoded protein involved in vacuolar ATPase formation, which has a crucial role in the modulation of the acidic microenvironment (Lu et al., 2021). Numerous research studies have found that dysregulation of V-ATPase is related to tumor growth and invasion (Whitton et al., 2018). Targeting V-ATPase could upregulate ER stress-related markers and inhibit mTOR signaling to exert anticancer effects (Kitazawa et al., 2017).

Survival analysis and ROC curve presented that the UPRRG risk signature demonstrated satisfactory predictive accuracy in both cohorts and that the low immune status in the high-risk group was strongly connected with poor prognosis. Moreover, our results identified that risk score was an independent prognostic factor for osteosarcoma patients. Subsequently, we performed a nomogram model to better predict the prognosis of osteosarcoma patients *via* risk score and clinical characteristics. Previous studies have reported several nomogram models to predict the prognosis of osteosarcoma (Qian et al., 2021). Li et al. built a nomogram model by autophagy-related genes with AUC values of 0.735 and 0.726 at 3 and 5 years, respectively (Li J. et al., 2021). Wen et al. developed a 3-gene nomogram model with 3-year and 5-year AUC values of 0.853 and 0.818, respectively (Wen et al., 2020). Wu et al. established a hypoxic nomogram model with an AUC value of only 0.73 (Wu et al., 2021). In our study, the constructed nomogram model had 3-year and 5-year AUC values of 0.93 and 0.90, respectively, which were consistently superior to other models. We presented that the UPRRG nomogram model could better predict the prognosis of patients with osteosarcoma than existing models. Collectively, these findings offered new options for personalized treatment and prognostic prediction of osteosarcoma.

Despite the many strengths of the current study, there are notable limitations. First, the UPRRG risk signature was constructed based on the TARGET and GEO databases, which may be biased due to the limited number of patients. Second, this study lacked some clinical information relevant to the prognosis of osteosarcoma, such as tumor pathological grade, which constrained clinical variables that can be incorporated into the nomogram model. A larger, multicenter, prospective clinical cohort will be needed to further evaluate the clinical merit of our findings in the future.

## Conclusion

Our study identified two molecular subtypes through consensus clustering based on prognosis-related UPRRGs, and the two subtypes exhibited different survival times and immune statuses. Functional analysis revealed that the UPRRG subtypes might influence the progression and prognosis of osteosarcoma patients through immune-related pathways. Moreover, a novel prognostic model based on UPRRGs was constructed and validated to better predict the prognosis of patients with osteosarcoma. We elucidated the important function of UPRRGs in the development and prognosis of osteosarcoma, shedding light on new insights for targeted therapy and clinical decision-making in patients with osteosarcoma.

## Data Availability

The original contributions presented in the study are included in the article/Supplementary Material; further inquiries can be directed to the corresponding author.
