# Supplementary figures and images for: Unfolded Protein Response–Related Signature Associates With the Immune Microenvironment and Prognostic Prediction in Osteosarcoma
Source: Front Genet. 2022 Jun 8;13:911346. doi: 10.3389/fgene.2022.911346 (PMC9214238; doi:10.3389/fgene.2022.911346)

**Figure S1.** The consistency values of different clustering groups.


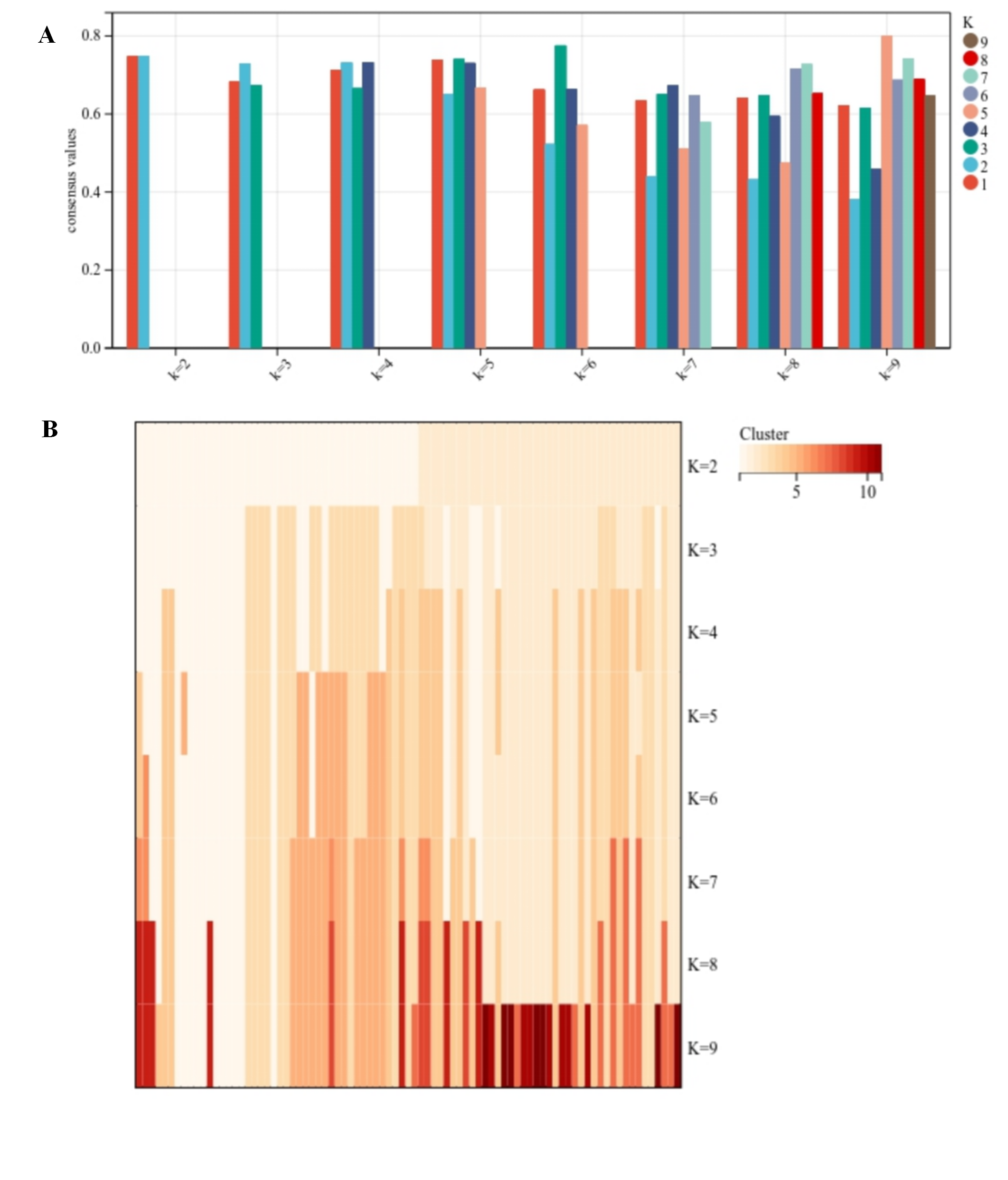

Supplement: Supplementary file 1 [file DataSheet1.docx]
